# From Static Stability to Dynamic Gait Governance: Differential Effects of Adapted Physical Activity and Tango-Based Therapy on Walking Distance and Plantar Load Redistribution in Parkinson’s Disease

**DOI:** 10.3390/sports14090387

**Published:** 2026-09-02

**Authors:** Raffaella R. R. Marzovillo, Gabriella D’Orsi, Paride Vasco, Giuseppe Cartagena, Donato Melchionda, Natalia Forte, Giuseppe Cibelli, Anna A. Valenzano

**Affiliations:** 1 Department of Clinical and Experimental Medicine, University of Foggia, 71100 Foggia, Italy; raffaella.marzovillo@unifg.it (R.R.R.M.); natalia_forte.591095@unifg.it (N.F.); giuseppe.cibelli@unifg.it (G.C.); anna.valenzano@unifg.it (A.A.V.); 2 Department of Humanities Education and Sport, Pegaso University, 80143 Naples, Italy; paride.vasco@unipegaso.it; 3 Sport Medicine Unit, Policlinic of Foggia, 71100 Foggia, Italy; giuseppe_cartagena.555937@unifg.it; 4 Department of Neurology, Policlinic of Foggia, 71100 Foggia, Italy; donatomelchionda@gmail.com

**Keywords:** adapted physical activity, Parkinson’s disease, exercise medicine, tango therapy, gait analysis, postural stability, plantar load, locomotor biomechanics, neurorehabilitation, preventive motor activity

## Abstract

Gait and balance impairments in Parkinson’s disease (PD) severely compromise mobility and independence. This exploratory randomized pilot study evaluated a multimodal Adapted Physical Activity (APA) protocol, a tango-based program (Riabilitango^®^, RT), and standard care regarding walking distance, static postural stability, joint range of motion, and plantar-load redistribution. Twenty-seven participants with PD (Hoehn and Yahr 1–3) were allocated to APA (*n* = 10), RT (*n* = 10), or Control (*n* = 7). The active groups completed 24 supervised sessions over 12 weeks. Static postural stability and treadmill-based walking distance were evaluated at baseline and post-intervention. Both active interventions showed favorable static sway profiles compared to Control, though between-group differences were non-significant. Crucially, APA demonstrated a significantly greater post-intervention improvement in walking distance than RT and Control. Exploratory analyses linked APA walking gains to adaptations in joint mobility and plantar-load redistribution. In conclusion, multimodal APA showed the clearest functional signal for improving walking distance in PD, whereas Riabilitango^®^ offered a complementary profile focused on sensorimotor integration and balance. These preliminary pilot findings warrant confirmation in larger prospective trials.

## 1. Introduction

Parkinson’s disease (PD) is a progressive neurodegenerative disorder marked by a wide range of motor impairments, such as bradykinesia, resting tremor, postural instability, and notable disturbances in gait and balance [1]. Among these impairments, freezing of gait and balance deficits substantially increase the risk of falls, resulting in severe injuries, persistent fear of falling, and eventual loss of independence [2,3].

Beyond these hallmark motor deficits, a wide array of non-motor symptoms is increasingly recognized as a major determinant of long-term disability. These encompass autonomic dysfunctions, sleep disorders, and prominent neuropsychiatric manifestations such as depression and anxiety [4,5,6]. Within the framework of the International Classification of Functioning, Disability and Health (ICF), recent evidence has shown that non-motor symptoms often correlate more strongly with reductions in Health-Related Quality of Life (HRQoL) than motor symptoms alone [7]. Epidemiologically, PD imposes a substantial and expanding global burden, affecting approximately 6.1 million individuals worldwide [8,9]. Neuropathologically, the hallmark of the disease is the abnormal accumulation of misfolded *α*-synuclein [8] and the progressive loss of dopaminergic neurons within the substantia nigra pars compacta [10,11], the functional severity of which is commonly staged clinically using the H&Y scale [12]. Pharmacological treatments, particularly levodopa, remain the gold standard for symptomatic management of PD; however, these therapies do not halt the underlying neurodegenerative process and are often associated with long-term motor complications [13]. Consequently, structured physical exercise is increasingly recommended core non-pharmacological strategy to support motor function, mobility, balance, and quality of life in individuals with PD [14]. Systematic reviews and clinical perspectives further support the inclusion of exercise as a central component of PD rehabilitation and long-term management [15,16].

Extensive research indicates that regular physical activity may facilitate activity-dependent neuroplasticity, increase brain-derived neurotrophic factor (BDNF) expression, and support motor learning and functional adaptation [17,18,19]. These mechanisms are presented as a theoretical framework and were not directly measured in the present study. Recent systematic reviews and network meta-analyses confirm the broad efficacy of various exercise modalities in improving functional capacity and motor symptoms in individuals with PD [15,20]. Nevertheless, the relative effectiveness of specific exercise modalities remains uncertain, especially when different interventions are evaluated within the same clinical and instrumental assessment framework.

Emerging evidence indicates that multimodal and sensorimotor interventions may be especially relevant for improving balance control, gait adaptability, and functional mobility in PD [16,18,21]. Balance-focused and postural-stability interventions have also demonstrated effects on mobility-related outcomes, although these effects vary depending on program content and patient characteristics [22,23,24]. Specifically, treadmill-based and task-specific locomotor training can serve as structured external pacing strategies, supporting gait rhythm, walking speed, and endurance [25,26].

In parallel, rhythm- and dance-based interventions have gained increasing attention in PD rehabilitation. Among these, Argentine tango and tango-derived therapeutic protocols have been associated with improvements in balance, gait, functional mobility, participation, and quality of life. These effects may be related to rhythmic auditory cueing, multi-directional stepping, backward walking, partner-mediated postural control, and social engagement [27,28,29,30,31]. Despite these advances, several important questions remain unresolved. Most previous studies have examined aerobic exercise and general exercise interventions in PD as broad rehabilitation categories [15,16,20]. Other research has focused specifically on treadmill-based locomotor training [25,26], while a separate body of evidence has investigated tango- and dance-based interventions [27,28,29,30,31]. However, limited data are available on direct comparisons between a multimodal APA program and a structured tango-based therapy such as Riabilitango^®^ within the same study design using objective instrumental outcomes.

This comparison holds clinical relevance because the two interventions are not interchangeable. APA focuses on aerobic conditioning, treadmill-based locomotor exposure, functional strengthening, balance tasks, and postural-load regulation, aligning with exercise-prescription principles for chronic neurological conditions [32,33,34]. In contrast, Riabilitango^®^ prioritizes rhythmic cueing, multi-directional stepping, partner interaction, sensory-motor integration, and movement confidence, consistent with evidence on tango-based rehabilitation in PD [27,28,29,30,31].

Another unresolved issue involves the relationship between static postural adaptations and dynamic walking capacity. Improvements in static posturographic measures do not necessarily translate into enhanced walking performance, particularly in PD, where bradykinesia, rigidity, impaired anticipatory postural adjustments, and altered gait automaticity may limit locomotor adaptation [1,6]. Recent quantitative gait studies underscore the importance of objective instrumental assessment for detecting PD-related alterations in lower-limb kinematics and locomotor biomarkers [35,36]. Thus, comparing static balance, walking distance, lower-limb range of motion, and plantar-load redistribution may help identify preliminary functional response profiles associated with different exercise modalities. In this context, the present exploratory randomized pilot-controlled intervention study was conducted to compare a multimodal APA program, a specialized tango-based therapy protocol (Riabilitango^®^, RT), and standard care in individuals with PD. The primary aim was to determine whether changes in walking distance corresponded with changes in static posturographic outcomes, defined as GA_D, and to investigate whether lower-limb range-of-motion variables and plantar-load redistribution were associated with walking performance. Due to the small sample size and exploratory nature of the study, correlation, regression, and coupling analyses were interpreted as hypothesis-generating observations rather than as evidence of causal biomechanical mechanisms or definitive treatment superiority.

## 2. Materials and Methods

### 2.1. Participants

This prospective controlled study evaluated the effects of RT and an APA program on gait kinematics, postural control, and stabilometric balance in patients with PD. Data were collected from February to July 2025 at several specialized facilities, including the Neurology Clinical Unit of the Foggia Polyclinic, the Sports Therapy Gym of the Sports Medicine Outpatient Department at the same Polyclinic, and the Libertango gym A.P.S. Participants were recruited voluntarily from the Neurology Clinical Unit outpatient service. Intervention group sizes ranged from two to three participants to ensure close supervision and safety. The study adhered to the Declaration of Helsinki and received approval from the Ethics Committee of the University Hospital Ospedali Riuniti of Foggia (approval code: Studio ETRMPEMP_2024; approval date: 17 October 2024). Written informed consent was obtained from all participants before enrollment.

### 2.2. Eligibility Criteria

Initial screening evaluated 30 individuals with PD, yielding a final sample of 27 participants. Inclusion criteria were as follows:

- a formal diagnosis of idiopathic PD confirmed by a neurologist according to established clinical criteria;
- disease severity between stage I and III on the Hoehn and Yahr scale [12];
- ability to walk independently without assistive devices for at least 10 min;
- clinical stability of the antiparkinsonian pharmacological regimen prior to enrollment, with no major medication changes during the intervention period.

Exclusion criteria included severe orthopedic comorbidities, uncorrected visual or vestibular deficits, unstable cardiovascular or metabolic conditions, or any concurrent medical condition contraindicating moderate-intensity physical exercise [37]. Participants were also excluded if they exhibited clinically relevant cognitive impairment, defined as a Mini-Mental State Examination (MMSE) score below 24/30, or if cognitive or neuropsychiatric symptoms limited their ability to understand instructions, provide reliable cooperation, or safely participate in the intervention.

Participants were instructed to maintain their standard pharmacological therapy and routine daily activities throughout the study and to refrain from initiating any new structured exercise programs outside the assigned intervention.

Baseline demographic, clinical, and instrumental characteristics are presented in Table 1. To address potential pre-intervention imbalances in small randomized samples, baseline comparisons across groups were included to enhance transparency. Due to the exploratory pilot design and small group sizes, *p*-values were interpreted descriptively and not used to establish formal baseline equivalence between groups.

At baseline, no statistically significant between-group differences were observed for age, body weight, sex distribution, H&Y stage, walking distance, Sway Area, Postural Load, or hip and knee ROM variables. BMI differed across groups, with a lower mean BMI in the APA group than in the RT and Control groups. This baseline BMI imbalance, together with the descriptive sex-distribution imbalance, was considered when interpreting post-intervention between-group differences.

### 2.3. Study Design and Allocation Procedure

This investigation was structured as an exploratory, prospective, randomized, parallel-group, pilot-controlled intervention study. Following eligibility screening and baseline assessment, participants were allocated to one of three study arms: a Control group that maintained standard activities of daily living, a Riabilitango^®^ tango-based therapy group (RT), or an APA group (APA).

Group allocation utilized a computer-generated random sequence with an intended 1:1:1 ratio. The randomization list was generated in R software (version 4.4.2; R Foundation for Statis-tical Computing, Vienna, Austria) by an investigator not involved in outcome assessment or intervention delivery. Due to the small sample size and exploratory design, final group sizes were slightly unequal. The three screened individuals who declined participation did so before randomization and before any treatment allocation was disclosed. Therefore, the smaller Control group did not result from post-allocation refusal of the no-intervention condition. Rather, the final group imbalance reflected the computer-generated allocation sequence applied to the 27 enrolled participants. No participant declined participation after allocation to the Control group. No formal allocation-concealment procedure was implemented, which should be considered when interpreting the results. Therefore, this study should be regarded as a pilot investigation intended to generate preliminary comparative evidence on the functional and biomechanical response profiles of two structured exercise interventions, rather than as a definitive efficacy trial.

The intervention phase lasted 12 weeks. Participants in the APA and RT groups completed 24 supervised sessions, delivered twice weekly for 60 min per session. Comprehensive instrumental evaluations of gait and posture were conducted at baseline (*T*_0_) and at the end of the 12-week intervention period (*T*_1_). Attendance was recorded at each supervised session, and adherence was calculated as the percentage of completed sessions out of the 24 scheduled.

Trial registration: The trial was retrospectively registered at ClinicalTrials.gov (NCT07736014) on 29 July 2026. Registration was completed after participant enrollment and study completion.

No formal a priori sample-size or power calculation was performed. The sample size was determined by the exploratory pilot nature of the study, the availability of eligible participants during the predefined recruitment period, and the organizational capacity required to deliver closely supervised APA and Riabilitango^®^ sessions. Accordingly, the study was designed to estimate preliminary functional and biomechanical response patterns and to inform future adequately powered trials, rather than to provide definitive efficacy estimates.

### 2.4. Interventions

All participants were instructed to maintain their usual antiparkinsonian pharmaco-logical regimen and routine daily activities for the duration of the study. Additionally, participants were required to avoid initiating any new structured exercise or rehabilitation programs outside of the assigned study condition. These measures were implemented to minimize contamination between groups and enhance the interpretability of between-group comparisons.

#### 2.4.1. Control Condition

Participants assigned to the Control group maintained their standard medical care and daily activities without receiving supervised APA or Riabilitango^®^ sessions throughout the 12-week study period. They were instructed to refrain from initiating new structured exercise programs, dance-based rehabilitation, physiotherapy, or other supervised motor-rehabilitation interventions during the observation period. Informal checks at follow-up assessed compliance with these instructions; however, no formal physical activity log was implemented. This limitation was considered in the interpretation of the control condition.

#### 2.4.2. Riabilitango^®^ Tango-Based Therapy Protocol (RT)

Each RT session began with a standardized 10 min warm-up targeting joint mobility and weight-shifting between the lower limbs. The central phase focused on dynamic postural control and multi-directional stepping, utilizing Argentine tango elements specifically adapted for individuals with PD. These elements included rhythmic walking synchronized to external auditory cues, pivoting movements, backward walking, and progressive directional changes. The final phase comprised partnered exercises intended to improve physical connection, somatosensory feedback, reactive balance, and movement confidence. All RT sessions were supervised and tailored to participants’ motor abilities and safety needs. This intervention structure aligns with previous evidence supporting tango-based and dance-based approaches for improving gait, balance, mobility, and participation in PD [27,28,29,30,31].

#### 2.4.3. Adapted Physical Activity Protocol

The APA program was developed in accordance with the FITT-VP principles established by the American College of Sports Medicine [35]. Each session began with a 5 min warm-up on a cycle ergometer, followed by aerobic conditioning on a treadmill. Treadmill intensity was adjusted to achieve a target heart rate of 60–70% of maximum heart rate (HRmax). Cardiovascular responses were monitored in real time using a Polar H10 chest strap (Polar Electro Oy, Kempele, Finland). The duration of treadmill walking was progressively increased from 20 to 30 min, after which participants engaged in functional resistance exercises, balance-oriented tasks, and a targeted stretching protocol. This proto-col was designed as a multimodal intervention that integrates aerobic, locomotor, strength, balance, and flexibility components, consistent with clinical exercise-prescription principles and recommendations for individuals with chronic neurological conditions [32,33,34].

### 2.5. Instrumental Postural, Stabilometric, and Gait Assessment

All instrumental evaluations were conducted while participants were in their self-reported “ON” medication state, following their usual antiparkinsonian pharmacological schedule. Although the interval between medication intake and testing was not strictly standardized, assessments were scheduled to coincide with each patient’s optimal motor response to therapy. When possible, baseline and post-intervention evaluations were performed at similar times of day and within comparable individual medication-response windows to reduce intra-subject variability associated with dopaminergic fluctuations. Investigators responsible for data processing and statistical analysis were blinded to group allocation. However, due to the behavioral nature of the interventions, neither participants nor exercise supervisors could be blinded to treatment assignment.

#### 2.5.1. Postural and Stabilometric Assessment (ProKin System)

Static and dynamic postural alignment and stabilometric balance were assessed using the ProKin 252^®^ system (TecnoBody S.r.l., Bergamo, Italy). The assessment protocol consisted of a 30 s static stabilometric test performed in the upright Romberg position with eyes open [38]. The primary variables measured were Sway Area (SEEO_A, mm^2^) and Postural Load distribution (PA_P.S.I.AVGtotal, %), which quantify weight-bearing distribution during static stance.

#### 2.5.2. Instrumental Gait Analysis

Kinematic and spatiotemporal gait parameters were collected using the TecnoBody Walker View 3.0 SCX treadmill (TecnoBody S.r.l., Bergamo, Italy), a device validated for measuring spatiotemporal and kinematic gait parameters [39]. After 2 min of familiarization, participants completed a continuous walking test at a self-selected speed. The primary functional outcome was walking distance (GA_D), measured in kilometers. Secondary kinematic outcomes included lower-limb range of motion (ROM). A comprehensive summary of all dataset variables, including their respective instrumental source systems, operational definitions, and units of measurement, is detailed in Table 2.

### 2.6. Study Design Quality Control

To enhance internal validity, all instrumental assessments were performed using a standardized protocol at both *T*_0_ and *T*_1_. Participants were instructed to maintain their regular pharmacological treatments and daily routines throughout the study period, and to refrain from starting any additional structured exercise programs beyond the assigned intervention. Attendance was documented for each supervised session, and adherence was calculated as the proportion of completed sessions out of the 24 scheduled. Safety was assessed by recording vital signs before and after each session, as well as by documenting any adverse events, falls, musculoskeletal complaints, cardiovascular symptoms, or interruptions to the intervention.

### 2.7. Statistical Analysis

Statistical analyses were conducted on a per-protocol basis. The final analytical population comprised participants who completed both baseline (*T*_0_) and post-intervention (*T*_1_) assessments. For those in the active intervention groups, inclusion in the per-protocol analysis also required meeting the predefined adherence threshold of at least 17 out of 24 supervised sessions. No imputation procedures were necessary, as no post-intervention outcome data were missing in the final analytical sample. Analyses were performed using R software, version 4.4.2 (R Foundation for Statistical Computing, Vienna, Austria).

Descriptive statistics are presented as mean ± standard deviation for continuous variables and as absolute frequency and percentage for categorical variables. Baseline comparability across groups was assessed using one-way ANOVA or Kruskal–Wallis tests for continuous variables, depending on distributional assumptions, and Fisher’s exact test or chi-square test for categorical variables, as appropriate. Due to the small sample size, baseline comparisons were interpreted descriptively and not considered evidence of group equivalence. Where relevant, standardized differences were also examined to identify clinically meaningful baseline imbalances.

The alpha level for statistical significance was set at *p <* 0.05, using two-tailed tests. Given the exploratory pilot design and the relatively large number of statistical comparisons relative to sample size, inferential results were interpreted alongside effect sizes, 95% confidence intervals, and clinical plausibility. Statistical significance alone was not considered sufficient evidence of definitive treatment efficacy or mechanism. Prior to inferential analyses, model assumptions were evaluated. Normality was assessed using the Shapiro–Wilk test and visual inspection of Q–Q plots, with particular attention to model residuals. Homogeneity of variance across groups was examined using Levene’s test. For ANCOVA models, the homogeneity-of-regression-slopes assumption was assessed before interpreting adjusted group effects. Because no a priori power calculation was performed, all inferential analyses were interpreted in the context of the feasibility-driven pilot design. Given the small sample size, inferential results were interpreted in conjunction with effect sizes, 95% confidence intervals, and graphical inspection of the data. The primary functional outcome was GA_D expressed in kilometers. A two-way mixed ANOVA was used to examine the effects of Group (APA, RT, Control), Time (*T*_0_, *T*_1_), and the Group × Time interaction on walking distance. The Group × Time interaction for GA_D was prespecified as the principal functional signal of interest in this exploratory analysis. When significant main or interaction effects were identified, post hoc between-group comparisons were conducted using Tukey’s honestly significant difference adjustment.

Between-group differences in changes in postural parameters were evaluated us-ing one-way ANOVA applied to delta values (∆ = *T*_1_ − *T*_0_). To control for baseline biomechanical asymmetries and postural-load differences, ANCOVA was performed on post-intervention Sway Area, with postural load included as a covariate. Effect sizes for ANOVA and ANCOVA models were reported as partial eta-squared (*η*^2^).

Between-group effect sizes for post-intervention walking distance and static sway area were calculated using Cohen’s d with 95% confidence intervals. Due to the small sample size, effect-size estimates were interpreted cautiously and primarily served to complement inferential statistics. Effect-size magnitude was interpreted according to Sawilowsky’s expanded descriptors: small (0.20), medium (0.50), large (0.80), very large (1.20), and huge (>2.0). Pearson correlation coefficients were used to explore associations between walking distance and lower-limb range-of-motion variables, as these variables were continuous and relationships were inspected graphically for approximate linearity and absence of influential outliers. Where appropriate, Spearman rank correlations were conducted as sensitivity analyses to verify the robustness of observed associations. To reduce the risk of type I error across multiple bivariate correlation tests, adjusted *p*-values were calculated using the Holm–Bonferroni procedure. Both unadjusted and adjusted *p*-values were reported for the principal exploratory correlation analyses. Due to the small sample size and multiple testing, secondary correlation findings were interpreted as hypothesis-generating rather than confirmatory.

Simple linear regression models were used to explore associations between selected biomechanical predictors and functional outcomes, including the relationship between joint ROM and GA_D, as well as the association between changes in postural load and changes in sway area. Regression diagnostics included inspection of residual plots and evaluation of influential observations. Subgroup-specific regression models were interpreted with particular caution, as each group included only 7 to 10 participants. Consequently, high *R*^2^ values were considered potentially unstable and were not used to infer causal mechanisms or definitive predictive models. These regression and delta-coupling analyses were interpreted as exploratory biomechanical associations and hypothesis-generating evidence, rather than as evidence of causality or definitive mechanisms of treatment response.

## 3. Results

### 3.1. Participant Flow, Adherence, and Safety

**Figure 1:**
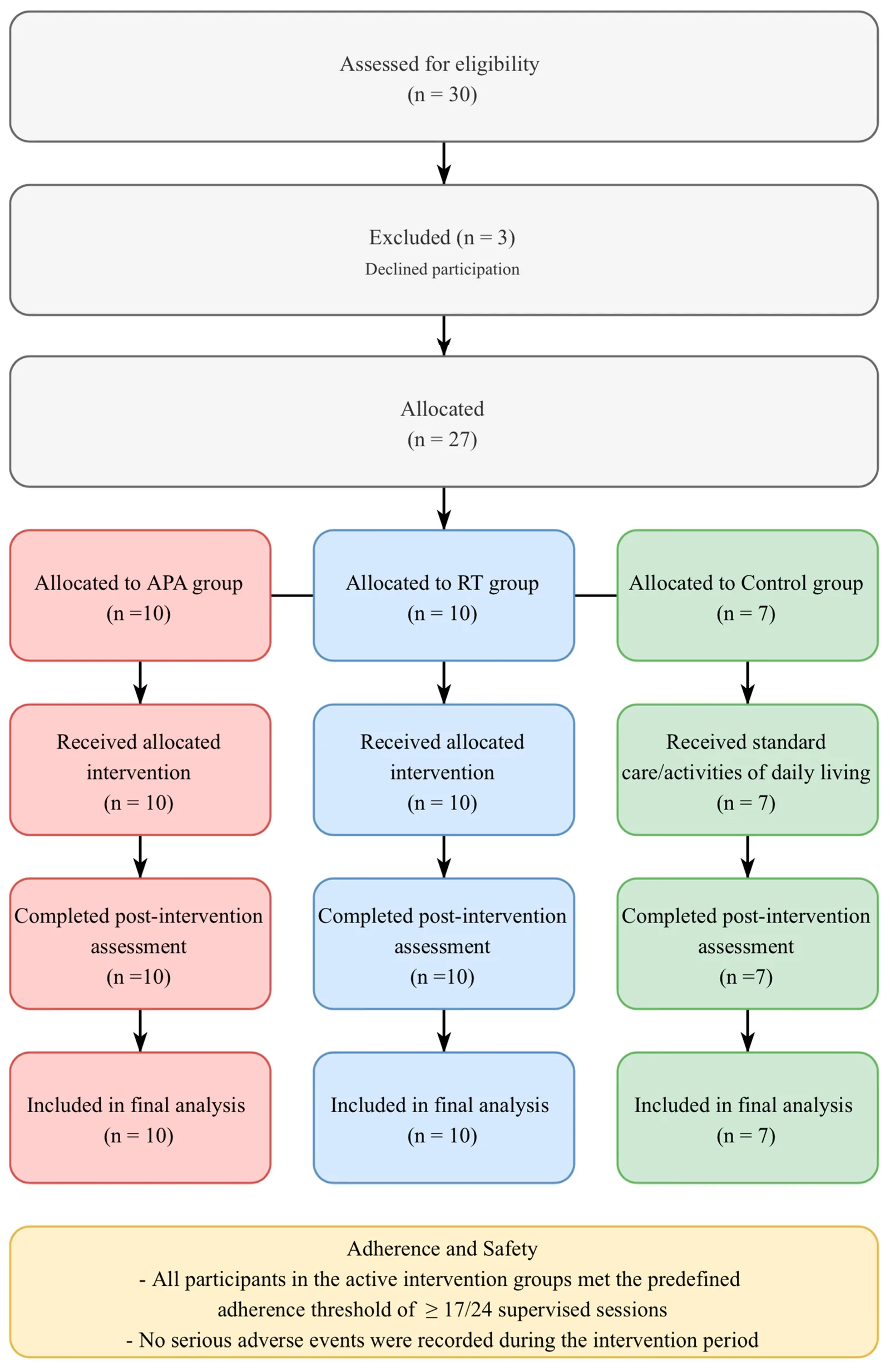
Participant flow diagram, allocation, and per-protocol final analysis population. Note: Downward arrows depict the sequential progression of participants through the trial stages. Color coding distinguishes the intervention groups and summary sections: grey boxes represent initial screening and overall allocation; pink boxes represent the Adapted Physical Activity (APA) group; blue boxes represent the Riabilitango^®^ (RT) group; green boxes represent the Control group; and the light yellow box summarizes adherence and safety monitoring.

Among the 30 individuals assessed for eligibility, three declined participation before randomization. The remaining 27 participants were randomized and allocated to APA (*n* = 10), RT (*n* = 10), or Control (*n* = 7). No post-allocation withdrawal occurred, and all randomized participants completed the post-intervention assessment and were included in the final per-protocol analysis (Figure 1).

All participants in the active intervention groups achieved the predefined adherence threshold of at least 17 out of 24 supervised sessions. The mean attendance was 21.8 ± 1.7 sessions in the APA group and 21.4 ± 1.9 sessions in the RT group, corresponding to adherence rates of 90.8 ± 7.1% and 89.2 ± 7.9%, respectively. Across the two active intervention groups, overall attendance was 21.6 ± 1.8 sessions, corresponding to an adherence rate of 90.0 ± 7.5%. No serious adverse events were recorded during the intervention period.

There were no falls, cardiovascular symptoms requiring interruption, or intervention-related serious musculoskeletal events reported during supervised sessions.

### 3.2. Baseline Characteristics and Pre-Intervention Comparisons

Baseline demographic, clinical, and instrumental characteristics are presented in Table 1. No statistically significant differences were observed at baseline for age, body weight, sex distribution, H&Y stage, walking distance, Sway Area, Postural Load, or lower-limb ROM variables. However, BMI differed significantly across groups (*p* = 0.040), with the APA group exhibiting lower mean values compared to the RT and Control groups. Although sex distribution did not differ statistically between groups, a descriptive imbalance was observed: the APA group included a higher proportion of male participants, while the Control group included a higher proportion of female participants. Due to the small sample size, these baseline characteristics were considered clinically relevant for interpretation, even in the absence of statistically significant differences. Therefore, baseline *p*-values were interpreted descriptively, with emphasis placed on identifying potential imbalances rather than establishing formal group equivalence.

### 3.3. Static Postural Stability and Balance Outcomes

To evaluate changes in static postural stability, a one-way ANOVA was performed on the delta scores (∆ = *T*_1_ − *T*_0_) for Sway Area (SEEO_A). The analysis did not reveal a statistically significant difference between the three groups (*F*(2, 24) = 0.886, *p* = 0.425, *η*^2^ = 0.069, and observed power = 0.203).

To further control for initial biomechanical asymmetries, an ANCOVA was performed on the post-intervention (*T*_1_) SEEO_A, using Postural Load as a covariate. The covariate did not show a significant main effect (*F*(1, 23) = 2.56, *p* = 0.123), and the main effect of the intervention group did not reach statistical significance (*F*(2, 23) = 0.72, *p* = 0.498).

While the omnibus ANOVA and ANCOVA models did not reveal statistically significant between-group differences, post-intervention effect-size estimates indicated favorable static sway profiles for both active interventions compared to standard care. Cohen’s d values demonstrated large effect sizes for SEEO_A in the APA versus Control comparison (*d* = 1.15) and in the RT versus Control comparison (*d* = 0.88). The APA versus RT comparison yielded a negligible effect size (*d* = 0.04), indicating broadly similar static sway profiles between the two active interventions. In summary, the static postural findings indicate favorable posturographic trends for both APA and RT, despite the lack of statistically significant results in the omnibus tests (Table 3).

### 3.4. Functional Gait Capacity and Walking Performance

**Figure 2:**
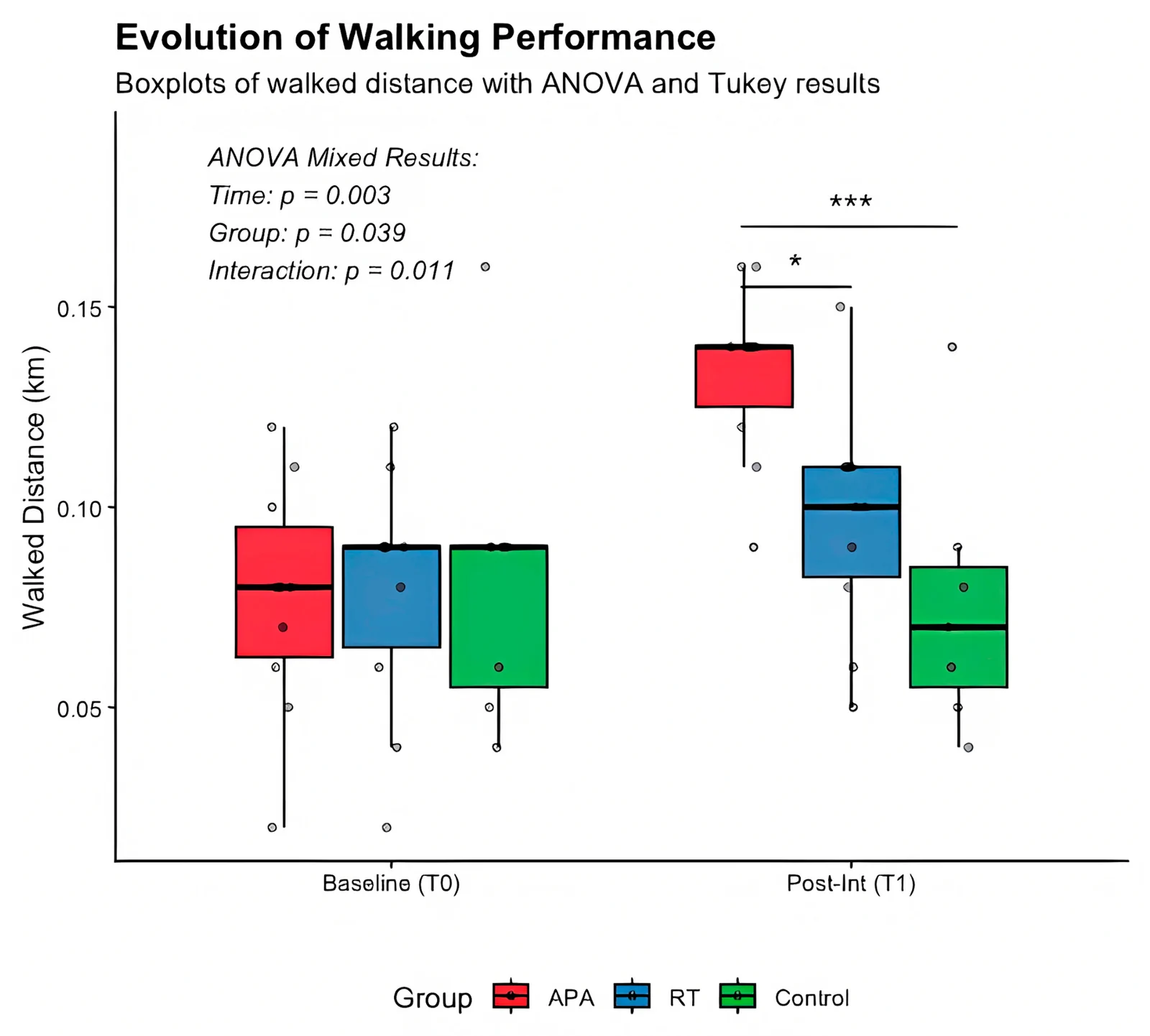
Mixed ANOVA boxplot illustrating the distribution and significant variations of GA_D (km) across APA, RT, and Control groups from baseline (T0) to post-intervention (T1). Asterisks indicate statistical significance levels for post hoc Tukey HSD pairwise comparisons at post-intervention: * *p* < 0.05 (APA vs. RT) and *** *p* < 0.001 (APA vs. Control).

A two-way mixed ANOVA was conducted to examine the effects of Group (APA, RT, Control) and Time, and their interaction on GA_D. The analysis revealed a significant main effect of Time (*F*(1, 48) = 9.64, *p* = 0.003), indicating overall changes in walking performance across the sample, as well as a significant main effect of Group (*F*(2, 48) = 3.45, *p* = 0.039). A statistically significant Group × Time interaction was observed (*F*(2, 48) = 4.95, *p* = 0.011), indicating that changes in GA_D over the 12-week period differed by intervention group (see Figure 2). This interaction represented the primary statistically supported functional finding of the study.

**Figure 3:**
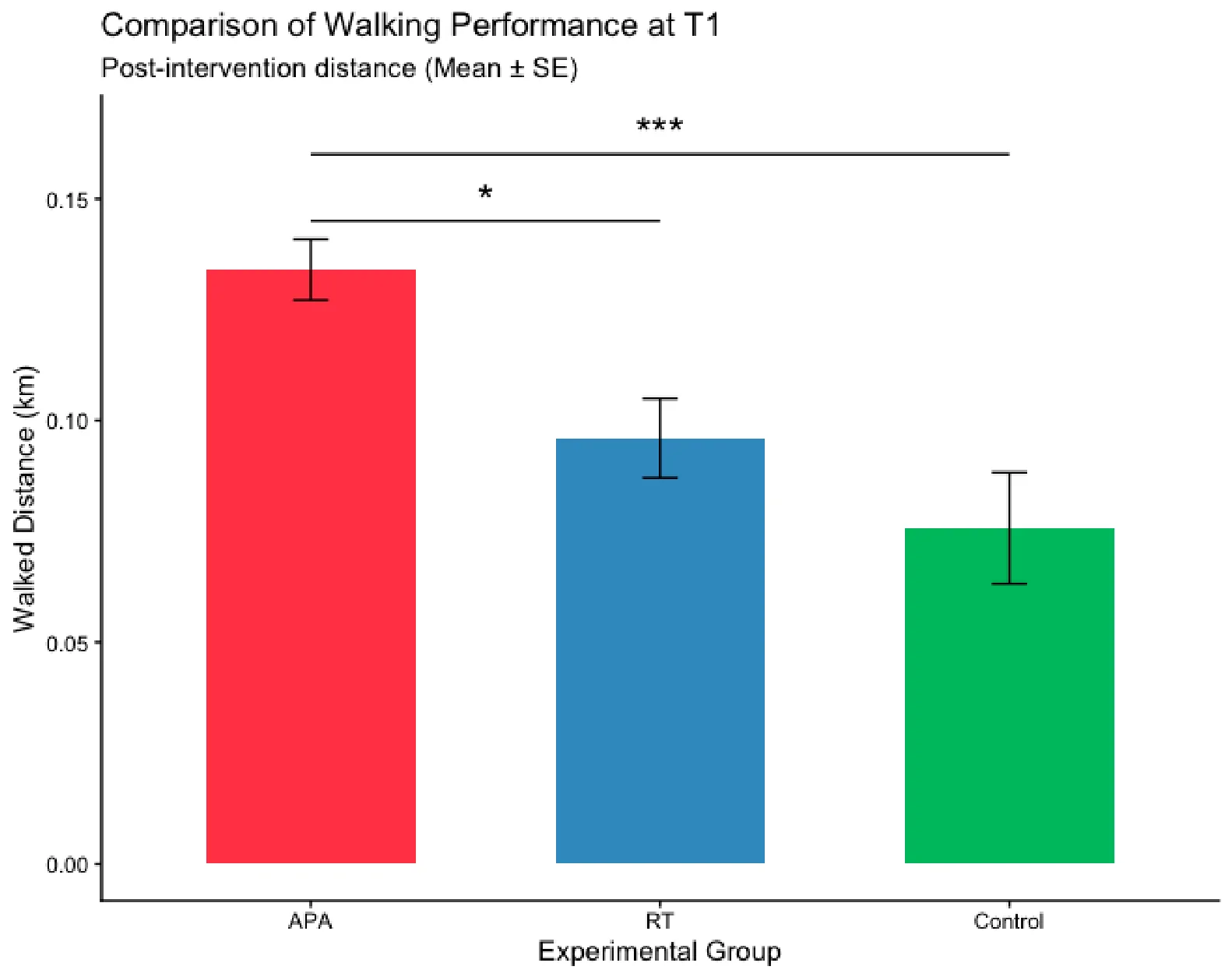
Pairwise post hoc comparisons (Tukey’s HSD) at post-intervention (*T*_1_), illustrating the greater walking distance achieved by the APA group compared to the RT and Control groups. Asterisks indicate statistically significant pairwise differences between groups at post-intervention (T1): * *p* < 0.05 (APA vs. RT) and *** *p* < 0.001 (APA vs. Control). Error bars represent mean ± standard error (SE).

Pairwise post hoc comparisons using Tukey’s HSD adjustment at *T*_1_ indicated that the APA group achieved a greater GA_D than both the Control group (mean difference = 0.058 km, SE = 0.015, *t* = 3.91, and *p* < 0.001) and the RT group (mean difference = 0.038 km, SE = 0.013, *t* = 2.81, and *p* = 0.019). No significant difference was observed between RT and Control at post-intervention. Therefore, APA exhibited the most distinct post-intervention walking-distance improvement, whereas RT was less clearly associated with improvement in this outcome. (see Figure 3).

These findings were further substantiated by effect-size analysis (see Table 3). The APA intervention demonstrated a large to huge observed effect compared to the Control group (*d* = 2.17) and a very large observed effect compared with the RT group (*d* = 1.50; 95% CI: [0.44, 2.57]). Effect-size estimates served as descriptive complements to inferential statistics. In contrast, the RT versus Control comparison showed a moderate observed effect size (*d* = 0.67), indicating a smaller and less consistent walking-distance improvement than that observed for APA. Collectively, the walking-distance results indicate that the multimodal APA protocol may have produced a stronger transfer to dynamic walking performance than RT or standard care. However, this between-group signal should be interpreted as preliminary rather than as definitive evidence of clinical superiority (see Table 3).

### 3.5. Exploratory Associations Among Walking Distance, Joint Range of Motion, and Postural-Load Redistribution

To explore the relationship between lower-limb kinematics and functional walking performance, bivariate correlation analyses were performed between GA_D and selected lower-limb ROM variables across the whole cohort. These analyses were considered exploratory because of the limited sample size and multiple statistical comparisons.

Positive correlations were identified between GA_D and lower-limb ROM variables within the analytical sample. Significant correlations were observed for Left Hip ROM (*r* = 0.283, *p* = 0.038), Right Hip ROM (*r* = 0.312, *p* = 0.021), Left Knee ROM (*r* = 0.366, *p* = 0.006) and Right Knee ROM (*r* = 0.380, *p* = 0.004) (see Table 4).

These results indicate that greater lower-limb joint excursion is associated with improved walking performance, with statistical robustness assessed using Holm-adjusted *p*-values. Although knee ROM variables demonstrated numerically stronger correlations than hip ROM variables, these differences should be interpreted with caution, as the analyses were not intended to establish a hierarchy of biomechanical determinants.

Simple linear regression models were applied to determine whether the association between selected ROM variables and walking distance varied across groups and time points.

**Figure 4:**
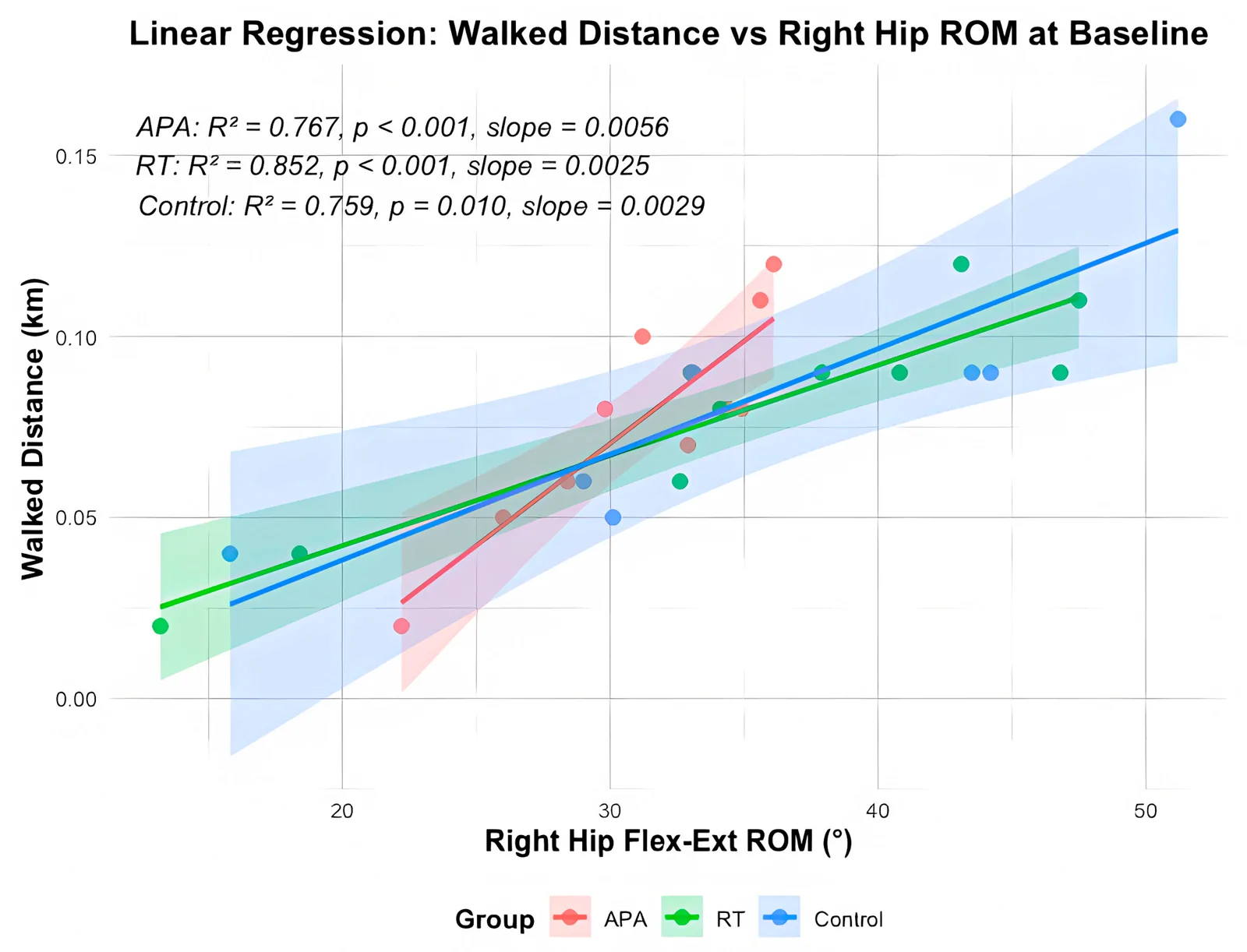
Linear regression analysis illustrating the relationship between Right Hip Flex-Ext ROM and Walking Distance (m) at baseline (T0) for APA (red), RT (green), and Control (blue) groups.

At baseline (*T*_0_), right hip flexion-extension ROM demonstrated positive associations with GA_D in all three groups. The coefficients of determination were high in this small (see Figure 4) (APA: *R*^2^ = 0.767, *p* < 0.001, slope = 0.0056; RT: *R*^2^ = 0.852, *p* < 0.001, slope = 0.0025; Control: *R*^2^ = 0.759, *p* = 0.010, slope = 0.0029), likely due to the limited number of participants per group (7^10^), rendering the *R*^2^ estimates unstable.

**Figure 5:**
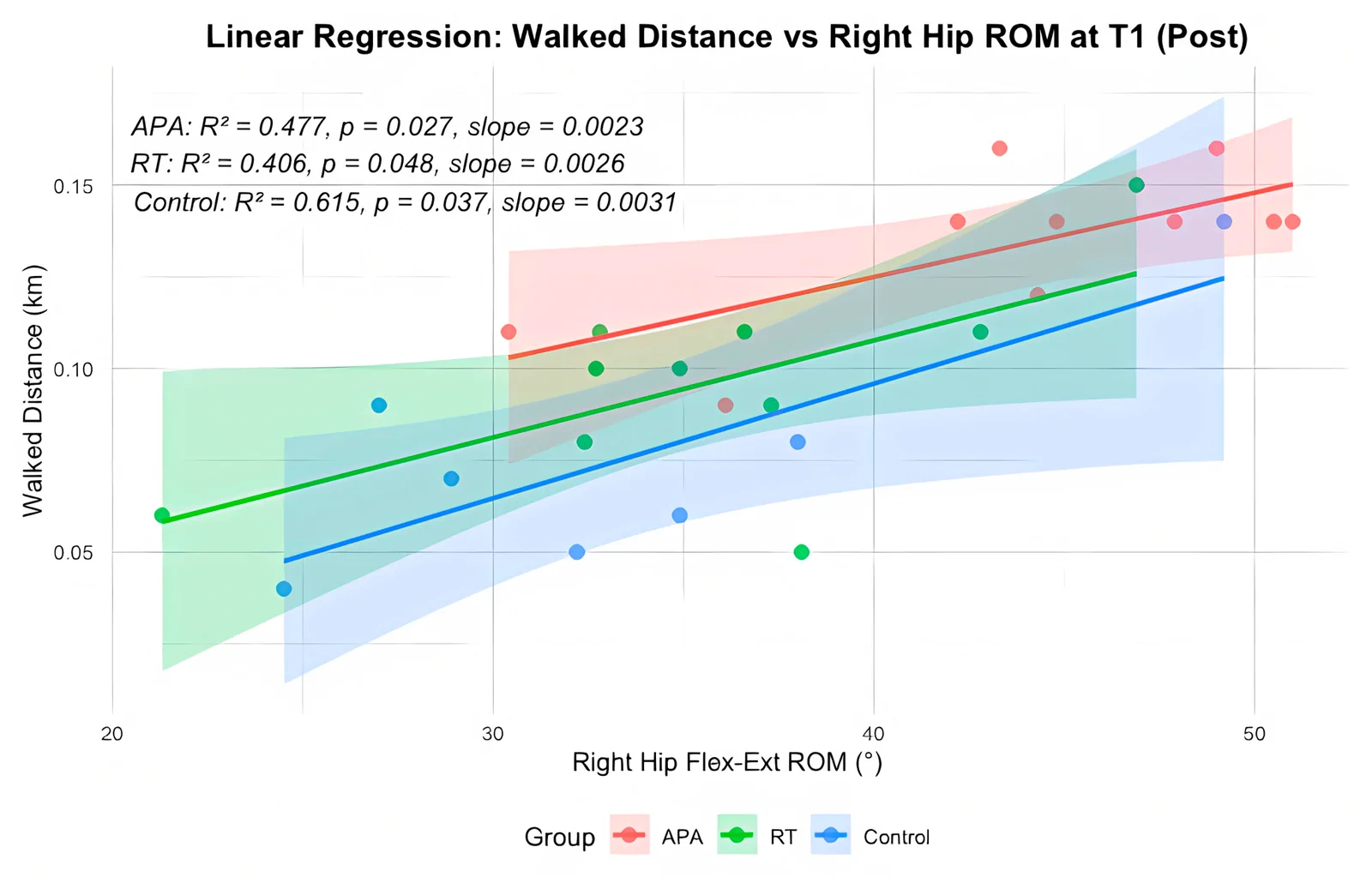
Linear regression analysis illustrating the relationship between Right Hip Flex-Ext ROM and Walking Distance (m) post-intervention (T1) for APA (red), RT (green), and Control (blue) groups.

Post-intervention (*T*_1_), the association between right hip ROM and GA_D was attenuated in the active groups (see Figure 5) (APA: *R*^2^ = 0.477, *p* = 0.027, slope = 0.0023; RT: *R*^2^ = 0.406, *p* = 0.048, slope = 0.0026), while the Control group continued to demonstrate a positive association (*R*^2^ = 0.615, *p* = 0.037, slope = 0.0031). This pattern suggests a potential change in the relationship between joint mobility and walking performance following active intervention.

A similar exploratory analysis was conducted for left knee flexion-extension ROM. At baseline, left knee ROM was associated with GA_D in the Control group (*R*^2^ = 0.731, *p* = 0.014, slope = 0.0033) and the RT group (*R*^2^ = 0.465, *p* = 0.030, slope = 0.0019), but not in the APA group (*R*^2^ = 0.266, *p* = 0.127). At *T*_1_, the association no longer statistically significant in the APA group (*R*^2^ = 0.010, *p* = 0.783) and RT group (*R*^2^ = 0.304, *p* = 0.098), while it remained significant in the Control group (*R*^2^ = 0.843, *p* = 0.003, slope = 0.0035). These findings indicate that the association between knee excursion and walking performance varied across groups and time points.

**Figure 6:**
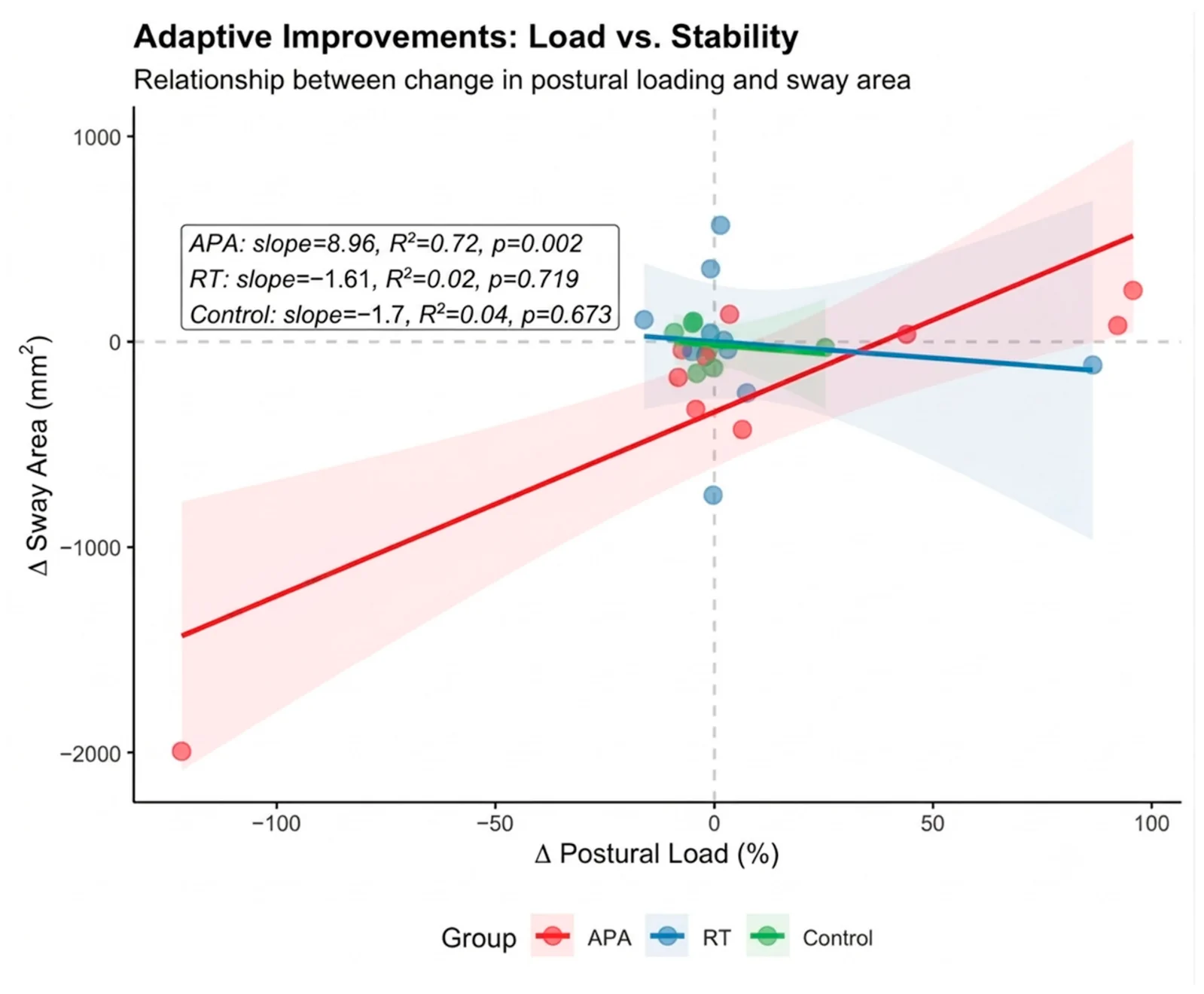
Linear regression models applied to delta scores (∆*T*_1_ − *T*_0_), illustrating the association between changes in postural-load distribution and sway area. A statistically significant relationship was observed exclusively in the APA group.

Finally, exploratory delta-coupling analyses were conducted to examine associations between changes in Total Postural Load (%) and changes in SEEO_A (mm^2^). In the APA group, changes in postural-load distribution were associated with changes in SEEO_A variation (slope = 8.96, *R*^2^ = 0.72, *p* = 0.002). This association was not observed in the RT group (slope = −1.61, *R*^2^ = 0.02, *p* = 0.719) or the Control group (slope = −1.70, *R*^2^ = 0.04, *p* = 0.673) (see Figure 6).

A sensitivity ANCOVA-based repeated-measures model was performed for GA_D with baseline BMI included as a covariate. The Group × Time interaction remained statis-tically significant after adjustment (F = 13.81, *p* < 0.001). Adjusted post hoc comparisons at T1 showed that the APA group retained a greater GA_D than Control (adjusted mean difference = 0.057 km, *p* < 0.001) and RT (adjusted mean difference = 0.034 km, *p* = 0.011). An additional exploratory model including both baseline BMI and sex as covariates yielded a similar pattern of results, with the APA group maintaining the highest adjusted post-intervention GA_D. However, given the small group sizes and uneven sex distribution, this model was interpreted as supportive rather than definitive. Overall, these analyses indicate potential relationships among GA_D, lower-limb range of motion, plantar-load redistribution, and static postural stability, thereby providing preliminary evidence to inform future mechanistic studies.

## 4. Discussion

### 4.1. Principal Findings and Interpretation

The primary objective of this study was to compare the effects of a multimodal APA protocol, RT Tango-based therapy, and standard care on postural stability, gait kinematics, plantar load redistribution, and functional walking capacity in patients with PD. The results provide preliminary evidence that distinct structured exercise modalities are associated with specific functional response profiles in PD. Among the measured outcomes, GA_D emerged as the most robust indicator of functional improvement in this sample. This interpretation aligns with the broader consensus that exercise is a clinically relevant component of PD management and rehabilitation [20,40,41]. Systematic reviews and meta-analyses further support the role of structured exercise in improving motor symptoms, mobility, and functional capacity in PD [15,19,20]. The principal statistically supported finding was a significant Group × Time interaction for GA_D. Post-intervention comparisons demonstrated that the APA group achieved greater GA_D than both the RT and Control groups. These results suggest that the multimodal APA protocol yielded the most pronounced improvement in dynamic walking performance within this pilot cohort.

In contrast, RT was less clearly associated with improvements in GA_D within the current outcome framework. This result does not indicate that RT lacks rehabilitative value. Instead, it suggests that the two active interventions may target different therapeutic dimensions. APA was more directly associated with GA_D improvement, while RT may be clinically relevant for enhancing rhythmic cueing, sensory-motor integration, partner-mediated balance control, motivation, and adherence [27,28,29,30,31]. Static postural outcomes demonstrated favorable effect-size profiles for both APA and RT compared to standard care. However, omnibus ANOVA and ANCOVA analyses did not reveal statistically significant between-group differences. This pattern is consistent with existing literature indicating that exercise can influence balance and postural control in PD, although effects may vary depending on the intervention and outcome measures used [22,23,24]. Correlation, regression, and delta-coupling analyses, conducted as secondary assessments, suggested potential relationships among joint mobility, plantar-load redistribution, static postural control, and walking performance.

### 4.2. Differential Functional Profiles of APA and Riabilitango^®^

A key contribution of this study is the direct comparison of two structured exercise approaches that differ markedly in their motor, sensory, and task-specific demands. This comparative perspective constitutes a significant element of novelty. While previous re-search has investigated general exercise and multimodal rehabilitation in PD [15,16,20], treadmill-based locomotor training [25,26], and tango- or dance-based interventions [27,28,29,30,31], limited data exist regarding how these distinct exercise modalities produce different functional and biomechanical response profiles within a single controlled framework. The most pronounced difference between interventions in this study was observed in GA_D, with APA demonstrating greater improvement compared to RT or standard care. This outcome may be attributed to the specific structure of the APA protocol, which integrates treadmill-based locomotor exposure, aerobic conditioning, functional strengthening, balance-oriented exercises, and postural-load regulation within a single intervention. This interpretation aligns with existing evidence supporting task-specific locomotor training and treadmill-based approaches for enhancing gait rhythm, walking speed, and endurance in PD [25,26], as well as with clinical exercise-prescription guidelines that recommend individualized multimodal training for chronic neurological conditions [32,33,34]. In contrast, RT may elicit a distinct adaptive profile. Riabilitango^®^ tango-based therapy incorporates rhythmic auditory cueing, multi-directional stepping, backward walking, partner-mediated postural adjustments, and social interaction. These components have been linked to improvements in balance, mobility, participation, and quality of life in previous tango- and dance-based PD studies [27,28,29,30,31]. Consequently, the lack of a statistically significant difference between RT and control for GA_D does not preclude a clinically meaningful RT effect in domains not fully reflected by GA_D alone. Collectively, these findings support the principle of goal-oriented exercise prescription in PD Individuals whose primary rehabilitation objective is to improve walking endurance and dynamic gait capacity may benefit from protocols emphasizing locomotor, aerobic, and task-specific components [16,25,26,32]. In contrast, those experiencing balance insecurity, freezing-related issues, reduced confidence, or poor adherence may derive greater benefit from rhythm- and dance-based interventions such as RT [27,28,29,30,31]. Although activity-dependent neuroplasticity provides a plausible theoretical framework for interpreting exercise-related improvements in PD [17,18,19], this study did not include direct neurophysiological assessments. Therefore, neuroplastic mechanisms should be considered only as a potential background explanation rather than as a conclusion supported by the present data. These findings indicate the need for further investigation of multimodal APA and tango-based interventions as potentially complementary, rather than competing, exercise strategies in PD.

### 4.3. Exploratory Biomechanical Associations

Exploratory correlation and regression analyses were conducted to investigate potential relationships among walking distance, lower-limb ROM, plantar-load redistribution, and static postural stability. These analyses aimed to identify biomechanical associations rather than to establish causality. Recent literature supports the use of quantitative gait and postural assessments, demonstrating that PD is associated with measurable alterations in spatiotemporal gait parameters, lower-limb kinematics, and objective locomotor biomarkers [35,36]. At baseline, walking distance demonstrated a positive association with selected lower-limb ROM variables, particularly hip and knee excursions. This observation is clinically plausible, as reduced joint excursion, bradykinesia, rigidity, and impaired postural adjustments are known contributors to altered walking performance in PD [1,2,3,4,5,6]. Recent gait-analysis literature further substantiates the relevance of ROM and kinematic parameters as objective descriptors of PD-related locomotor impairment [35,36]. However, the subgroup regression models produced high coefficients of determination despite the small sample sizes, indicating that these R^2^ values should be interpreted as unstable exploratory estimates. Following the 12-week intervention, the relationship between selected ROM variables and walking distance changed in the active intervention groups. This pattern may indicate a modified association between joint mobility and walking performance as a result of structured exercise. Similarly, delta-coupling analyses indicated that changes in postural-load distribution were associated with changes in sway-area variation in the APA group. In contrast, this association was not observed in the RT or Control groups. These findings suggest a potentially relevant relationship between load redistribution and postural-control adaptation following APA Overall, the biomechanical analyses provide preliminary evidence that may inform future research. Subsequent studies should prospectively define biomechanical endpoints, recruit larger and more balanced samples, apply appropriate multiplicity control, and integrate clinical outcomes with neurophysiological and instrumented gait measures. These steps are necessary to determine whether the associations observed in this study represent reproducible exercise-related adaptations in PD.

### 4.4. Clinical Significance and Exercise-Prescription Implications

The present findings provide preliminary evidence supporting the inclusion of structured exercise as a key element of multidisciplinary care in PD [20,40,41]. Consistent with previous systematic reviews and network meta-analyses, exercise programs incorporating aerobic, coordinative, balance, and functional components have the potential to improve motor symptoms, mobility, and functional capacity in individuals with PD [15,20,24]. Rather than establishing a definitive hierarchy of interventions, these results support an exercise-prescription approach tailored to distinct functional response profiles. An important consideration arising from these findings is the clinical interpretation of changes in walking distance. In this sample, post-intervention between-group differences favored APA by approximately 0.058 km compared to Control and 0.038 km compared to RT, corresponding to 58 m and 38 m, respectively. These changes may have implications for daily mobility, walking autonomy, and functional reserve in patients with PD, especially when walking impairment leads to reduced independence and increased fear of falling [2,3,7]. The GA_D outcome in this study was obtained using an instrumented treadmill-based assessment and should not be considered directly equivalent to a conventional corridor-based six-minute walk test. Published estimates of the minimal clinically important difference for six-minute walk distance in adult pathological populations provide relevant clinical context, although these estimates vary by population and methodology [42]. For individuals with parkinsonism, Steffen and Seney reported an MDC95 of approximately 82 m for the six-minute walk test; however, this threshold applies to a clinical field test rather than the WalkerView-derived treadmill outcome used in this study [43]. Within this context, the magnitude of the APA-related walking-distance signal appears clinically plausible, but its clinical significance should be evaluated using standardized functional walking tests and patient-centered outcomes. From an exercise-prescription perspective, these findings support a goal-oriented approach rather than prioritizing a hierarchy of interventions. Multimodal APA may be particularly suitable when the primary therapeutic objective is to enhance walking endurance, dynamic gait capacity, and functional mobility, as it incorporates locomotor, aerobic, strengthening, and balance-related components [16,25,26,32]. Riabilitango^®^ tango-based therapy may be especially beneficial when rehabilitation goals include rhythmic cueing, sensory-motor integration, partner-mediated balance control, movement confidence, social participation, and sustained adherence to exercise [27,28,29,30,31]. The clinical implication is that APA and RT should be regarded as potentially complementary exercise strategies. Patients whose primary limitation is reduced walking endurance or dynamic gait capacity may benefit from interventions emphasizing locomotor and aerobic components [16,25,26,32]. In contrast, patients experiencing balance insecurity, freezing-related concerns, diminished confidence, or challenges with motivation may respond more favorably to rhythm- and dance-based approaches [27,28,29,30,31]. Future research should directly evaluate this goal-oriented model by integrating instrumented biomechanical outcomes with standardized clinical measures, including corridor-based six-minute walk distance, freezing of gait assessment, fall history, balance confidence, quality of life, and patient-reported mobility outcomes. Such an approach would enable future trials to determine whether statistically significant changes in walking distance correspond to clinically meaningful improvements in daily functioning [37,41,42,43].

## 5. Study Limitations

Several limitations of the present study warrant consideration.

1. The total sample size was relatively small (N = 27), with limited numbers in each study arm. This limitation reduces statistical power, restricts generalizability to the broader PD population, and necessitates cautious interpretation of subgroup analyses, effect-size estimates, and exploratory regression models. Consequently, the biomechanical and delta-coupling findings should be regarded as hypothesis-generating rather than confirmatory. Notably, the high coefficients of determination observed in certain subgroup regression models should be interpreted with caution, as estimates derived from 7 to 10 participants per group are inherently unstable and may be influenced by single observations.
2. Although group allocation utilized a computer-generated random sequence, no formal allocation-concealment procedure was implemented. This methodological limitation may have introduced potential selection bias and should be considered when evaluating the internal validity of the findings. Additionally, the final group sizes were slightly unequal, reflecting the exploratory nature and limited scale of the study.
3. The analysis was conducted on a per-protocol basis. Although all participants included in the final analysis completed both baseline and post-intervention assessments, and all participants in the active groups met the predefined adherence thresh-old, the absence of an intention-to-treat (ITT) analysis limits the generalizability of the findings to broader clinical settings where adherence and dropout rates may vary.
4. The control condition constitutes an additional methodological limitation. Although participants in the Control group were instructed not to initiate new structured exercise or rehabilitation programs during the observation period, compliance was monitored informally, and no structured physical-activity log was maintained. As a result, unsupervised changes in habitual physical activity cannot be entirely excluded.
5. A notable limitation involves baseline demographic imbalances across groups, particularly regarding sex distribution and BMI. Baseline comparisons across groups are reported to enhance transparency concerning potential pre-intervention imbalances. BMI differed among groups, with the APA group predominantly male and the Control group comprising a higher proportion of female participants. As sex- and BMI-related differences may influence PD phenotype, gait characteristics, postural control, and responsiveness to exercise interventions, these imbalances should be considered when interpreting the findings. However, given the small sample size, non-significant baseline comparisons should not be regarded as evidence of equivalence between groups.
6. Although sensitivity analyses adjusting for baseline BMI, and exploratorily for sex, supported the same overall pattern observed in the primary analysis, the small and uneven group sizes limit the precision of covariate-adjusted estimates.
7. Although all instrumental assessments were conducted during the self-reported “ON” medication state, the exact interval between antiparkinsonian medication intake and instrumental testing was not strictly standardized. This limitation may have introduced residual variability related to dopaminergic motor fluctuations and should be more rigorously controlled in future studies.
8. The study included multiple exploratory analyses, such as ANOVA/ANCOVA models, post hoc comparisons, correlation analyses, subgroup-specific regressions, and delta-coupling models. Although corrections for multiple testing were applied where appropriate, the number of analyses relative to the small sample size increases the risk of false-positive findings. Therefore, secondary statistical results should be interpreted as exploratory rather than confirmatory.
9. The study primarily focused on instrumental posturographic, gait, ROM, and plantar-load outcomes. While this focus is a strength from a biomechanical perspective [35,36,39], future trials should incorporate additional clinical endpoints, such as disease-specific motor scales, freezing of gait assessments, fall history, balance confidence, quality of life, and patient-reported outcomes, to better assess the clinical relevance of the observed instrumental changes [37,41]. Additionally, GA_D in the present study was measured using an instrumented treadmill-based assessment and should not be considered directly comparable to corridor-based six-minute walk distance. Future studies should include standardized clinical walking tests to determine whether changes in instrumented GA_D correspond to clinically meaningful improvements in everyday mobility [42,43,44].
10. The study was not prospectively registered. Although the trial was retrospectively registered at ClinicalTrials.gov (NCT07736014) on 29 July 2026, registration occurred after participant enrollment and study completion. This constitutes a significant methodological limitation regarding transparency and prospective documentation of trial procedures. Larger, prospectively registered randomized controlled trials with allocation concealment, balanced recruitment, intention-to-treat analysis, extended follow-up, adequate multiplicity control, and integrated clinical, neurophysiological, biomechanical, and patient-reported endpoints are required to confirm and expand upon these exploratory findings.

## 6. Conclusions

This exploratory randomized pilot-controlled intervention study provides preliminary evidence that a multimodal APA protocol may yield a more pronounced improvement in walking distance compared to RT or standard care in individuals with PD. APA was associated with the greatest improvement in GA_D, while RT demonstrated a distinct intervention profile. The observed improvement in GA_D with APA aligns with the locomotor, aerobic, and task-specific components of the protocol [16,25,26,32]. In contrast, the RT profile is consistent with previous evidence supporting tango- and dance-based interventions for rhythmic cueing, sensory-motor integration, balance confidence, social engagement, and adherence in PD [27,28,29,30,31]. Both active interventions demonstrated favor-able effect-size profiles for static postural outcomes; however, between-group ANOVA and ANCOVA analyses for SEEO_A did not reach statistical significance. Biomechanical analyses generated hypotheses concerning potential associations among GA_D, lower-limb ROM, plantar-load redistribution, and postural stability. This interpretation is supported by the established relevance of quantitative gait and kinematic assessments in PD [35,36]. These findings support the clinical relevance of further investigation into multimodal APA and tango-based interventions as potentially complementary exercise strategies for PD, with intervention selection tailored to individual functional goals and motor phenotype. This goal-oriented approach aligns with current PD rehabilitation recommendations and exercise-prescription principles [32,33,34,41]. Given the pilot design, lack of allocation concealment, use of per-protocol analysis, and absence of prospective trial registration, these results should be validated in larger, prospectively registered randomized trials that incorporate baseline-balanced recruitment, intention-to-treat analysis, extended follow-up, and integrated clinical, neurophysiological, biomechanical, and patient-reported outcomes.

## Figures and Tables

**Table 1:** Baseline demographic, clinical, and instrumental characteristics by intervention group (*T*_0_).

Characteristic	Total (*N* = 27)	APA (*N* = 10)	RT (*N* = 10)	Control (*N* = 7)	*p*-Value
**Demographics & Clinical**					
Age (years)	70 ± 9	70 ± 7	68 ± 8	71 ± 12	0.825 ^*a*^
Weight (kg)	75 ± 14	70 ± 12	79 ± 18	75 ± 11	0.412 ^*a*^
BMI (kg/m^2^)	27.6 ± 4.5	24.8 ± 3.3	29.6 ± 5.5	28.8 ± 2.7	0.040 ^*a*^
**Sex**, *n* (%)					0.128 ^*b*^
– Female	8 (30%)	1 (10%)	3 (30%)	4 (57%)	
– Male	19 (70%)	9 (90%)	7 (70%)	3 (43%)	
**Hoehn & Yahr Stage**, *n* (%)					0.542 ^*b*^
– Stage 1	3 (11%)	0 (0%)	1 (10%)	2 (29%)	
– Stage 1.5	2 (7%)	2 (20%)	0 (0%)	0 (0%)	
– Stage 2	5 (19%)	3 (30%)	1 (10%)	1 (14%)	
– Stage 2.5	11 (41%)	3 (30%)	5 (50%)	3 (43%)	
– Stage 3	6 (22%)	2 (20%)	3 (30%)	1 (14%)	
**Functional & Postural (** *T*_0_ **)**					
Gait Distance (GA_D, km)	0.079 ± 0.032	0.077 ± 0.029	0.079 ± 0.031	0.083 ± 0.040	0.936 ^*a*^
Sway Area (SEEO_A, mm^2^)	670 ± 530	630 ± 770	370 ± 310	170 ± 80	0.199 ^*a*^
Postural Load (PA_P.S.I., %)	17.0 ± 32.3	6.7 ± 44.1	20.5 ± 28.0	26.8 ± 11.4	0.429 ^*a*^
**Joint Kinematics/ROM (** *T*_0_ **)**					
Right Hip Max ROM (°)	19.6 ± 6.0	18.8 ± 4.4	19.1 ± 7.8	21.3 ± 5.3	0.678 ^*a*^
Left Hip Max ROM (°)	18.4 ± 5.9	17.7 ± 4.7	16.9 ± 7.3	21.5 ± 4.7	0.263 ^*a*^
Right Knee Max ROM (°)	46.8 ± 8.1	46.8 ± 6.0	44.3 ± 9.7	50.4 ± 7.8	0.313 ^*a*^
Left Knee Max ROM (°)	47.3 ± 8.0	47.9 ± 6.6	46.7 ± 9.3	47.3 ± 9.2	0.947 ^*a*^

Note: Data are presented as Mean ± Standard Deviation for continuous variables and *n* (%) for categorical variables. Bold text denotes main category headers. *a* Calculated using One-way ANOVA. *b* Calculated using Fisher’s exact test. Abbreviations: APA = Adapted Physical Activity; RT = Riabilitango^®^ tango-based therapy; Control = Control Group; H&Y = Hoehn and Yahr; BMI = Body Mass Index; ROM = Range of Motion; GA_D = Walking Distance; PA_P.S.I. = Mean Postural Load (%); and SEEO_A = Center of Pressure Ellipse Area (Eyes Open, mm^2^). Given the exploratory pilot design, *p*-values were interpreted descriptively and were not used to establish formal baseline equivalence.

**Table 2:** Operational Definitions, System Sources, and Measurement Units of Dataset.

Variable Code	System/Assessment	Full Parameter Name	Measurement Unit/Coding
Group	Demographics	Intervention Group Assessment	1 = APA; 2 = RT; 3 = Control
*t*	Timeline	Assessment Time Point	1 = Baseline (*T*_0_); 2 = Post-intervention (*T*_1_)
SEEO_A	ProKin (Stabilometry)	Center of Pressure Ellipse Area (Eyes Open)	mm^2^
SEEO_P	ProKin (Stabilometry)	Center of Pressure Perimeter (Eyes Open)	mm
SEEO_V	ProKin (Stabilometry)	Mean Sway Velocity (Eyes Open)	mm/s
SEEC_A	ProKin (Stabilometry)	Center of Pressure Ellipse Area (Eyes Closed)	mm^2^
SEEC_P	ProKin (Stabilometry)	Center of Pressure Perimeter (Eyes Closed)	mm
SEEC_V	ProKin (Stabilometry)	Mean Sway Velocity (Eyes Closed)	mm/s
DE_TSI	ProKin (Dynamic)	Total Stability Index	Degrees (°)
PA_P.S.I.AVGtotal	ProKin (Postural)	Mean Postural Load/Weight-Bearing Distr.	%
GA_D	Walker View (Gait)	Total Walking Distance Covered	km
VO CoG	Walker View (Gait)	Vertical Oscillation of the Center of Gravity	cm
ROM_H_R_MAX	Walker View (Kinematics)	Right Hip Maximum Range of Motion	Degrees (°)
ROM_H_L_MAX	Walker View (Kinematics)	Left Hip Maximum Range of Motion	Degrees (°)
ROM_H_R_MAX	Walker View (Kinematics)	Right Knee Maximum Range of Motion	Degrees (°)
ROM_K_L_MAX	Walker View (Kinematics)	Left Knee Maximum Range of Motion	Degrees (°)

Note: APA = Adapted Physical Activity; RT = Riabilitango^®^ Tango-based therapy; Control = Control Group; and ROM = Range of Motion.

**Table 3:** Effect Size Analysis (Cohen’s *d*) for Postural Stability (Sway Area) and Functional Walking Outcomes at Post-Intervention (*T*_1_).

Variable Comparison	Outcome Measure	Cohen’s d	Effect Magnitude	Descriptive Clinical Interpretation
**Static Postural Stability**				
APA vs. Control	Sway Area (SEEO_A)	1.15	Large	Favorable observed static sway profile
RT vs. Control	Sway Area (SEEO_A)	0.88	Large	Favorable observed static sway profile
APA vs. RT	Sway Area (SEEO_A)	0.04	Negligible	Comparable static sway profiles
**Functional Walking Outcomes**				
APA vs. Control	Walking Distance (GA_D)	2.17	Huge	Large observed walking distance difference
APA vs. RT	Walking Distance (GA_D)	1.50	Very Large	Large observed difference favoring APA
RT vs. Control	Walking Distance (GA_D)	0.67	Medium	Moderate observed walking distance difference

Note Effect magnitude thresholds are interpreted according to Sawilowsky’s expanded descriptors for Cohen’s *d*: small (≥0.20), medium (≥0.50), large (≥0.80), very large (≥1.20), and huge (≥2.0). Abbreviations: APA = Adapted Physical Activity; RT = Riabilitango^®^ Tango-based therapy; Control = Control Group; SEEO_A = Center of Pressure Ellipse Area (Eyes Open); GA_D = Walking Distance.

**Table 4:** Exploratory bivariate Pearson correlations between walking distance and lower-limb range of motion (ROM) across the whole cohort.

ROM Variable ^a^	Pearson *r*	Raw *p*-Value	Holm *p*-Value	Interpretation
Left Hip ROM	0.283	0.038	0.042	Positive association
Right Hip ROM	0.312	0.021	0.042	Positive association
Left Knee ROM	0.366	0.006	0.018	Positive association
Right Knee ROM	0.380	0.004	0.016	Positive association

^a^ All ROM variables are evaluated in bivariate correlation against 6 min walking test distance. Note: Correlations were calculated across the analytical sample and interpreted as exploratory. Holm-adjusted. *p*-values are reported to address multiplicity across the four bivariate correlation tests. ROM = range of motion.

## Data Availability

The data supporting the findings of this study are available from the corresponding author upon reasonable request.
